# Metabolic burden-based clinical-radiological model for predicting postoperative recurrence of hepatitis B-related hepatocellular carcinoma

**DOI:** 10.1186/s13244-025-02183-3

**Published:** 2026-01-05

**Authors:** Beixuan Zheng, Heqing Wang, Yuyao Xiao, Fei Wu, Chun Yang, Ruofan Sheng, Mengsu Zeng

**Affiliations:** 1 Department of Radiology, Zhongshan Hospital, Fudan University, Shanghai, China; 2 Department of Radiology, Zhongshan Hospital (Xiamen), Fudan University, Xiamen, China; 3 Shanghai Institute of Medical Imaging, Shanghai, China; 4 Department of Cancer Center, Zhongshan Hospital, Fudan University, Shanghai, China

**Keywords:** Metabolic abnormalities, Hepatocellular carcinoma, Hepatitis B, Magnetic resonance imaging

## Abstract

**Objectives:**

To establish a metabolic burden-based clinical-radiological model for predicting postoperative recurrence in hepatitis B virus (HBV)-related hepatocellular carcinoma (HCC) at Barcelona Clinic Liver Cancer (BCLC) stages 0-A.

**Materials and methods:**

This retrospective multi-center study included HBV-related HCC (BCLC 0-A) undergoing curative surgery. Metabolic burden was defined as the cumulative number of metabolic abnormalities. Trend test assessed dose-dependent relationship. Predictors were identified via univariate and multivariate Cox regression analyses, and a nomogram was developed. The model underwent internal validation (5-fold, 100 times cross) and external validation. Performance was evaluated using *C*-index, calibration curves, and decision curve analysis.

**Results:**

The internal and external cohorts consisted of 363 patients (55.9 ± 10.7 years, 295 males) and 74 patients (55.5 ± 10.2 years, 55 males). Recurrence risk increased by 1.53 times (*p* = 0.049) and 1.64 times (*p* = 0.018) for patients with 2 and 3–4 metabolic abnormalities (*p*trend = 0.022). Independent predictors included tumor burden score > 2.4 (HR = 2.40, *p* = 0.003), metabolic abnormalities ≥ 2 (HR = 1.49, *p* = 0.023), aspartate transaminase/alanine transaminase ratio > 1 (HR = 1.51, *p* = 0.012), albumin-bilirubin grade 2 (HR = 1.70, *p* = 0.020), arterial rim enhancement (HR = 1.87, *p* = 0.002) and mosaic appearance (HR = 1.55, *p* = 0.033). C-indices for predicting 2- and 5-year recurrence were 0.728 (95% CI: 0.726–0.729) and 0.674 (95% CI: 0.673–0.675) in training sets, 0.716 (95% CI: 0.711–0.720) and 0.657 (95% CI: 0.653–0.660) in internal validation sets, and 0.710 (95% CI: 0.602–0.855) and 0.683 (95% CI: 0.594–0.798) in external cohort. The model showed higher predictive efficacy (*p* < 0.001 for all) and better clinical net benefit compared to BCLC and CNLC staging systems in the very early/early-stage of HCCs.

**Conclusion:**

The metabolic burden-based clinical-radiological model effectively predicts postoperative recurrence in HBV-related HCC.

**Critical relevance statement:**

Patients with HBV-related HCC who have two or more coexisting metabolic abnormalities may have a higher risk of postoperative recurrence. The metabolic burden-based clinical-radiological model is valuable in predicting postoperative recurrence

**Key Points:**

- Metabolic abnormalities were dose-dependently related to the risk of postoperative recurrence.
- The clinical-radiological model showed well-predictive efficacy in validation cohorts.
- The clinical-radiological model displayed higher efficacy compared to existing staging systems for the very early/early-stage of HCCs.

**Graphical Abstract:**

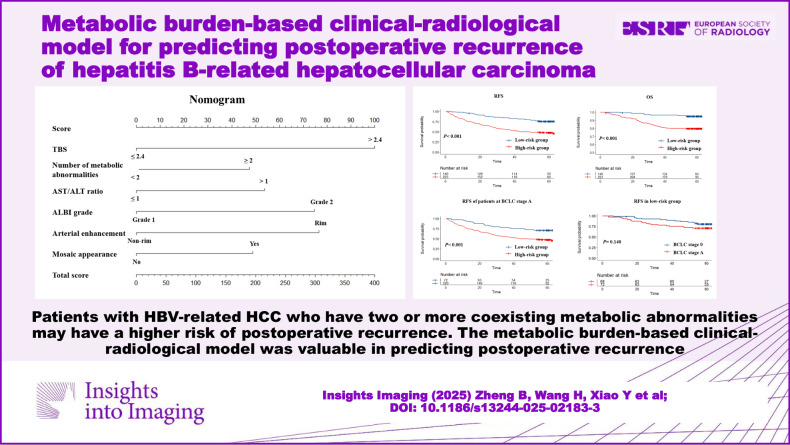

## Introduction

Primary liver cancer is the sixth most common cancer in the world, and hepatocellular carcinoma (HCC) is the most common type of primary liver cancer, accounting for about 90% of all cases [1]. Chronic hepatitis B virus (HBV) infection remains the predominant risk factor for HCC in China due to the persistently high prevalence [2]. According to the Barcelona Clinic Liver Cancer (BCLC) staging system, very early-stage HCC (stage 0) is defined as a single tumor ≤ 2 cm without macrovascular invasion or extrahepatic spread, while early-stage HCC (stage A) is defined as patients with a single tumor or up to three nodules, each ≤ 3 cm, without macrovascular invasion or extrahepatic metastasis [3]. Liver resection is the preferred curative treatment for patients at BCLC stages 0-A, but the prognosis is not ideal, with a high recurrence rate of 50–70% within five years [4].

Although conventional staging systems (e.g., BCLC and the China liver cancer (CNLC)) are valuable for treatment selection, they consider mostly the number and size of lesions, limiting precise recurrence risk stratification [5–7] This limitation is compounded for BCLC by its development in a cohort mainly consist of hepatitis C and alcoholic liver disease, which distinct from HBV-endemic populations [8, 9]. Meanwhile, growing evidence supports the value of MRI in surveillance, diagnosis, and treatment assessment of HCC, which can provide more prognostic information beyond just the size and number of lesions. Previous studies have noted that certain features from MRI indicated a heightened risk of recurrence for HCC, which may be related to aggressive biological behavior, and preoperative MRI-based risk stratification was not inferior to postoperative pathology in predictive efficacy [10, 11].

In recent years, an increasing number of studies have suggested that cancer is also a “metabolic disease” [12]. Tumor cells undergo metabolic reprogramming to accommodate rapid growth in environments lacking oxygen and nutrients [13]. The inducers of metabolic reprogramming include both intrinsic cellular factors and external factors such as obesity, diabetes, and so forth [13–15]. Insulin resistance, a key aspect of metabolic syndrome, emerges as a mechanistic pathway that could potentially drive cancer, including HCC [16, 17]. Furthermore, metabolic disorders lead to increased oxidative stress, which induces genetic mutations through oxidative DNA damage, leading to carcinogenesis [18]. Current studies have indicated that metabolic abnormalities are associated with the development and progression of HCC in patients with chronic hepatitis B [19–22]. Importantly, this metabolic burden (defined as the count of metabolic abnormalities, including overweight or obesity, elevated blood pressure, elevated blood glucose, and dyslipidemia) exhibits a dose-dependent relationship with HCC risk [23–26]. While the influence of metabolic abnormalities on postoperative recurrence and the existence of dose-dependent relationships have not been reported till now.

Therefore, the purpose of this study was to explore the impact of metabolic abnormalities and the dose-dependent burden on postoperative recurrence in HBV-related HCC at BCLC stages 0-A, and further to establish a metabolic burden-based clinical-radiological model for recurrence prediction.

## Materials and methods

This retrospective study was conducted according to the guidelines of the Declaration of Helsinki. It was approved by the Institutional Ethics Committee (approval no. B2024-240R), and the requirement for written informed consent was waived.

### Patients

This retrospective study enrolled adult patients (≥ 18-years-old) with HBV-related HCC at BCLC stages 0-A, who underwent curative-intent liver resection (R0 resection) at Zhongshan Hospital, Fudan University, from January 2018 to December 2018 as the internal cohort. Inclusion criteria were: (1) Pathologically confirmed HCC; (2) HBV-related HCC (HCC diagnosed in patients with either positive hepatitis B surface antigen or a documented history of chronic HBV infection); (3) BCLC stage 0 or A; (4) complete preoperative MRI data. Patients meeting the same inclusion criteria at Zhongshan Hospital, Fudan University, from January 2017 to February 2017, and its Xiamen branch from January 2018 to December 2021, were enrolled as the external cohort. Exclusion criteria were:(1) Incomplete clinical data; (2) co-infection with other chronic hepatitis or history of schistosomiasis infection; (3) received any antitumor treatments before; (4) interval between preoperative MRI examination and surgery more than one month; (5) unqualified MRI images not meeting diagnostic requirements; (6) history of other malignant tumors.

**Fig. 1:**
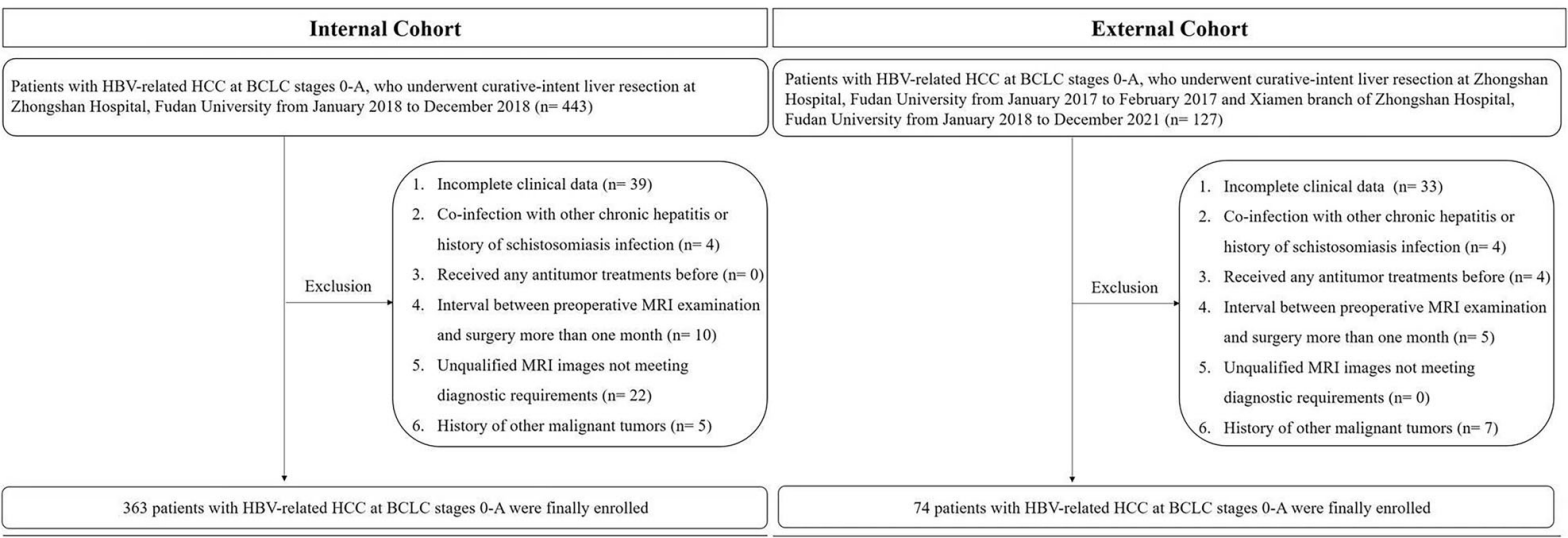
Flowchart of the study population. BCLC, Barcelona Clinic Liver Cancer; HBV, hepatitis B virus; HCC, hepatocellular carcinoma

Finally, 363 patients with HBV-related HCC at BCLC stages 0-A were enrolled as an internal cohort (295 males and 68 females, mean age: 55.9 ± 10.7 years), and 74 patients were enrolled as an external validation cohort (55 males and 19 females, mean age: 55.5 ± 10.2 years). The flowchart was presented in Fig. 1.

### Magnetic resonance examination

MR imaging was acquired using 1.5- or 3.0-Tesla MR scanners (Magnetom Aera, Siemens Healthcare; Prisma, Siemens Healthcare). The conventional magnetic resonance protocol evaluated in this study included T1-weighted in-phase and opposed-phase gradient echo sequence, transverse T2-weighted fast spin-echo sequence, and diffusion-weighted imaging (DWI) with *b* values of 0, 50, and 800 s/mm^2^. Dynamic imaging was performed with a T1-weighted fat-suppressed sequence. Gadopentetate dimeglumine (Magnevist; Bayer HealthCare) was intravenously administered at a rate of 2 mL/s for a dose of 0.1 mmol/kg. The arterial phase acquisition was automatically triggered when the contrast agent reached the ascending aorta, and the portal venous phase (70–90 s) and delayed phase (160–180 s) were acquired subsequently. Detailed sequence parameters are listed in Supplementary Table 1.

### Image analysis

All MRI images were independently evaluated by two board-certified abdominal radiologists (with 13 and 15 years of experience) who were blinded to the final diagnosis. Prior to this study, both observers received over 2 months of training using the Liver Imaging Reporting and Data System (LI-RADS) v2018 for lesion evaluation. For cases with discrepancies, the two observers reached a consensus through joint review. If a consensus could not be achieved, a final decision was made by a third senior abdominal radiologist. The following characteristics were investigated mainly based on the LI-RADS v2018 diagnostic algorithm [27]: (1) number of lesion; (2) location (adjacent to porta hepatis or not): adjacent to porta hepatis was defined as a lesion less than 1 cm away from the hepatic veins, portal veins, main branches of the biliary system, or inferior vena cava; (3) maximum diameter; (4) tumor burden score (TBS): calculated as TBS^2^ = (number of lesion)^2^ + (maximum diameter)^2^ [28]; (5) margin (regular or irregular); (6) signal homogeneity (homogenous, heterogenous), mainly evaluated on T2-weighted images; (7) arterial enhancement pattern (rim or non-rim); (8) non-peripheral washout; (9) arterial peritumoral enhancement: defined as detectable crescent or polygonal shaped enhancement surrounding the border on the arterial phase images, which becomes isointense during the delayed phase; (10) enhancing capsule (intact, incomplete or deficient); (11) mosaic appearance; (12) intratumoral necrosis; (13) intratumoral hemorrhage. For patients with multiple lesions, the biggest one was evaluated.

### Clinical and pathological data

The preoperative clinical features and postoperative pathological data were collected. All clinical indices and scores were obtained within one week before the surgery. The baseline metabolic abnormalities included: overweight or obesity (defined as body mass index [BMI] ≥ 24 kg/m^2^ based on Chinese criteria [29]), elevated blood pressure (including hypertension and high-normal blood pressure [30]), elevated blood glucose (including diabetes and prediabetes [31]), and atherogenic dyslipidemia (including hypertriglyceridemia and low high-density lipoprotein cholesterol [32]) (Supplementary Table 2).

### Study endpoint and follow-up

The primary endpoint of this study was time to recurrence. Recurrence was defined as intrahepatic or extrahepatic neoplasms observed by CT, MRI, ultrasound, or pathological confirmation. The secondary endpoints were recurrence-free survival (RFS) and overall survival (OS). Follow-up data were retrospectively collected from electronic medical records. According to institutional protocol, follow-up included abdominal contrast-enhanced MRI/CT, chest CT, and serologic tumor marker assessments. Patients were followed up at the outpatient clinic every 3 months for the first 2 years and every 6 months thereafter, with telephone contacts as supplements. The planned follow-up duration was at least 5 years or until recurrence, death, or loss to follow-up (no contact for more than 12 months). Median follow-up times were 60.3 months (IQR: 58.1–61.5 months) and 65.0 months (IQR: 38.9–83.9 months) for the internal and external cohorts, respectively.

### Statistical analyses

In this study, statistical analyses were conducted using the R language (version 4.2.3) or SPSS software (version 26.0). Statistical power analysis was performed using PASS 2011 (version 21.0.3). All statistical tests were two-sided, and *p* < 0.05 was considered significant.

Continuous variables with a normal distribution were presented as mean ± standard deviation (SD), while those with a skewed distribution were presented as median (interquartile range [IQR]). The normality of distribution was tested using the Shapiro-Wilk test.

Multivariable Cox regression was used to adjust for confounding factors, with adjusted hazard ratios (aHR) and 95% confidence intervals (CI) recorded. To explore potential nonlinear relationships between variables and the risk of recurrence, a restricted cubic spline with three knots after adjusting for confounding factors was used to draw smooth hazard ratio (HR) curves. A trend test was performed to assess whether a dose-dependent relationship existed [33].

Interobserver agreement was assessed using the interclass correlation coefficient (ICC) for continuous variables and kappa statistics for categorical variables. Variables with *p* < 0.20 in the univariate Cox regression were tested for collinearity by calculating the tolerance (TOL) and variance inflation factor (VIF). TOL < 0.1 and/or VIF > 10 indicated significant collinearity. After excluding variables with collinearity, the remaining variables were entered into multivariate regression with stepwise backward elimination using Akaike Information Criterion (AIC). The model constructed was internally validated using a 5-fold, 100-times cross-validation. Nomograms visualized the constructed model. The optimal cutoff of the risk score was determined based on the maximum Youden index. The cumulative incidence curves were used to compare the cumulative incidence rate of recurrence between the low- and high-risk groups. Kaplan–Meier curves were used to compare RFS and OS between different groups, with differences tested using the Log-rank method. Calibration curves were used to assess consistency, with bootstrap methods (*B* = 500) used for correction. Time-dependent *C*-index curves were utilized to illustrate the predicted efficacy at different time points. Decision curves were employed to assess the clinical net benefit. The generalizability of this model was further evaluated in the external cohort based on the optimal cutoff determined during model construction. The predictive performance of the BCLC and CNLC staging systems was evaluated as follows: each staging system was treated as an ordinal variable and entered as a single covariate into separate Cox regression models. The predictive accuracy of these models was assessed using the same evaluation framework applied to our clinical-radiological model.

Post hoc statistical power analysis was performed via a test for receiver operating characteristics using PASS 2021. For the internal cohort, the statistical power > 0.99 (alpha = 0.05, total sample size = 363, percent in group 1(+) = 0.50, area under curve 1 = 0.70). For the external cohort, the statistical power was 0.872 (alpha = 0.05, total sample size = 74, percent in group 1(+) = 0.50, area under curve 1 = 0.70).

## Results

### Baseline clinical characteristics

A total of 363 patients with HBV-HCC were ultimately enrolled as the internal cohort. Among them, 71 patients (19.6%) were at BCLC stage 0, and 292 patients (80.4%) were at BCLC stage A. Meanwhile, 74 patients were enrolled as the external validation cohort, including 12 patients (16.2%) at BCLC stage 0 and 62 patients (83.8%) at BCLC stage A. Baseline demographic characteristics are presented in Table 1.

**Table 1:** Baseline characteristics between the internal cohort and the external validation cohort

Variables		Internal cohort (*n* = 363)	External validation cohort (*n* = 74)	*p*
Age, mean ± SD, years		55.9 ± 10.7	55.5 ± 10.2	0.745
Gender	Male, *n* (%)	295 (81.3)	55 (74.3)	0.173
	Female, *n* (%)	68 (18.7)	19 (25.7)	
BCLC	Stage 0, *n* (%)	71 (19.6)	12 (16.2)	0.504
	Stage A, *n* (%)	292 (80.4)	62 (83.8)	
CNLC	Stage Ia, *n* (%)	249 (68.6)	52 (70.3)	0.777
	Stage Ib, *n* (%)	114 (31.4)	22 (29.7)	
Lesion number	1, *n* (%)	346 (95.3)	73 (98.6)	0.189
	2–3, *n* (%)	17 (4.7)	1 (1.4)	
Maximum diameter, mean ± SD, cm		4.2 ± 2.9	4.3 ± 3.0	0.799
Log AFP, median (IQR), ng/mL		1.23 (0.60, 2.23)	1.43 (0.67, 2.27)	0.250
ALT	> 50 U/L, *n* (%)	64 (17.6)	13 (17.6)	0.990
	≤ 50 U/L, *n* (%)	299 (82.4)	61 (82.4)	
AST	> 40 U/L, *n* (%)	73 (20.1)	13 (17.6)	0.616
	≤ 40 U/L, *n* (%)	290 (79.9)	61 (82.4)	
Total bilirubin	> 20.4 μmol/L, n (%)	54 (14.9)	5 (6.8)	0.063
	≤ 20.4 μmol/L, *n* (%)	309 (85.1)	69 (93.2)	
Albumin	< 35 g/L, *n* (%)	9 (2.5)	3 (4.1)	0.450
	≥ 35 g/L, *n* (%)	354 (97.5)	71 (95.9)	
Platelet count	< 100 × 10^9^/L, *n* (%)	45 (12.4)	9 (12.2)	0.955
	≥ 100 × 10^9^/L, *n* (%)	318 (87.6)	65 (87.8)	
Prothrombin time	> 13 s, *n* (%)	24 (6.6)	5 (6.8)	0.964
	≤ 13 s, *n* (%)	339 (93.4)	69 (93.2)	
Child–Pugh	A, *n* (%)	360 (99.2)	73 (98.6)	0.525
	B, *n* (%)	3 (0.8)	1 (1.4)	
Cirrhosis, *n* (%)		195 (53.7)	41 (55.4)	0.791
Number of metabolic abnormalities	< 2, *n* (%)	144 (39.7)	38 (51.4)	0.063
	≥ 2, *n* (%)	219 (60.3)	36 (48.6)	
Resection extent	Minor, *n* (%)	31 (8.5)	9 (12.2)	0.325
	Major, *n* (%)	332 (91.5)	65 (87.8)	
Intraoperative blood loss, median (IQR), mL		100 (100–200)	100 (100–200)	0.544
Antiviral therapy, *n* (%)		142 (39.1)	23 (31.0)	0.194
Grade	1, *n* (%)	6 (1.7)	1 (1.4)	0.018
	2, n (%)	239 (65.8)	61 (82.4)	
	3–4, *n* (%)	118 (32.5)	12 (16.2)	
MVI	0, *n* (%)	183 (50.4)	44 (59.4)	0.301
	1, *n* (%)	125 (34.4)	19 (25.7)	
	2, *n* (%)	55 (15.2)	11 (14.9)	

*ALT* alanine aminotransferase, *AST* aspartate aminotransferase, *BCLC* Barcelona Clinic Liver Cancer, *CNLC* the China Liver Cancer, *IQR* interquartile range, *MVI* microvascular invasion

### Metabolic abnormalities and risk of postoperative recurrence

The median follow-up time for the internal cohort was 60.3 months (IQR: 58.1–61.5 months). The 1-year, 2-year, and 5-year RFS rates were 83.7%, 73.5% and 57.1%, respectively. Latent nonlinear associations were observed between various metabolic indicators and risk of recurrence after adjusting for the number of lesions, maximum diameter, HBV deoxyribonucleic acid load, antiviral therapy, liver function, and cirrhosis (Supplementary Fig. 1). Overall, the risk of postoperative recurrence tended to increase with higher levels of diastolic blood pressure, BMI, fasting blood glucose, and plasma triglycerides, and decrease with higher levels of plasma high-density lipoprotein cholesterol. Multivariate Cox regression indicated that elevated blood glucose was an independent risk factor (HR = 1.45, 95% CI: 1.02–2.06, *p* = 0.040) (Table 2).

**Table 2:** Impact of metabolic abnormalities on postoperative recurrence

Variables	Univariate analyses	*p*	Multivariate analyses	*p*
	HR (95% CI)	*p*	aHR (95% CI)	*p*
Overweight/obese	1.08 (0.78–1.48)	0.642	1.29 (0.92–1.81)	0.146
Elevated blood pressure	1.36 (0.96–1.92)	0.087	1.42 (0.95–1.02)	0.088
Elevated blood glucose	1.37 (0.99–1.89)	0.055	1.45 (1.02–2.06)	0.040
Atherogenic dyslipidemia	0.92 (0.66–1.28)	0.605	1.13 (0.79–1.60)	0.514

Multivariate analyses were adjusted for the number of lesions, maximum diameter, HBV deoxyribonucleic acid load, antiviral therapy, liver function, and cirrhosis

*CI* confidence interval, *HR* hazard ratio

In the internal cohort, at least two types of metabolic abnormalities were present in 219 patients (60.3%): 101 patients (27.8%) had two, 84 patients (23.1%) had three, and 34 patients (9.4%) had four metabolic abnormalities. Among patients with metabolic abnormalities, 57 patients (41.9% of patients with elevated blood glucose) received antidiabetic therapy, 162 patients (69.2% of patients with elevated blood pressure) received antihypertensive medications, and 16 patients (11.8% of patients with atherogenic dyslipidemia) received lipid-modifying agents. Compared to patients with 0-1 metabolic abnormality, the risk of recurrence for patients with 2 and 3–4 metabolic abnormalities increased to 1.53 times (95% CI: 1.00–2.34, *p* = 0.049) and 1.64 times (95% CI: 1.09–2.48, *p* = 0.018), respectively, with a trend test *p* value of 0.022.

### Postoperative recurrence prediction model construction and internal validation

Table 3 summarizes the results of univariate and multivariate Cox regression analyses. Supplementary Table 3 summarizes the consistency of the assessment of MRI features among researchers. As shown in Supplementary Table 4, there was no significant collinearity among features with *p* < 0.20. TBS > 2.4 (HR = 2.40, 95% CI: 1.34–4.30, *p* = 0.003), number of metabolic abnormalities ≥ 2 (HR = 1.49, 95% CI: 1.06–2.11, *p* = 0.023), aspartate transaminase (AST)/alanine transaminase (ALT) ratio > 1 (HR = 1.51, 95% CI: 1.09–2.08, *p* = 0.012), albumin–bilirubin score (ALBI) grade 2 (HR = 1.70, 95% CI: 1.09–2.66, *p* = 0.020), arterial rim enhancement (HR = 1.87, 95% CI: 1.26–2.80, *p* = 0.002), and mosaic appearance (HR = 1.55, 95% CI: 1.04–2.31, *p* = 0.033) were identified as independently associated with the risk of recurrence.

**Table 3:** Univariate and multivariate Cox regression analyses for predicting postoperative recurrence

Variables		Univariate Cox analyses		Multivariate Cox analyses	
		HR (95% CI)	*p*	HR (95% CI)	*p*
Age, years old	≤ 55				
	> 55	1.11 (0.81–1.53)	0.525		
Gender	Female				
	Male	1.43 (0.91–2.25)	0.119		
TBS	≤ 2.4				
	> 2.4	3.00 (1.81–4.97)	< 0.001	2.40 (1.34–4.30)	0.003
Antiviral therapy		0.86 (0.62–1.20)	0.370		
HBV-DNA,	< 2				
kIU/mL	≥ 2	1.38 (1.00–1.92)	0.052		
Number of metabolic	< 2				
Abnormalities	≥ 2	1.30 (0.93–1.82)	0.125	1.49 (1.06–2.11)	0.023
Cirrhosis		0.96 (0.70–1.32)	0.797		
Log DCP, ng/mL	≤ 3				
	> 3	2.17 (1.54–3.04)	< 0.001		
AST/ALT ratio	≤ 1				
	> 1	1.51 (1.10–2.08)	0.011	1.51 (1.09–2.08)	0.012
ALBI grade	1				
	2	1.67 (1.08–2.58)	0.022	1.70 (1.09–2.66)	0.020
Location	Adjacent to the porta hepatis	1.29 (0.89–1.89)	0.182		
Margin	Regular				
	Irregular	5.87 (2.86–12.04)	< 0.001		
Signal homogeneity	Homogenous				
	Heterogenous	1.38 (1.00–1.91)	0.047		
Arterial enhancement	Non-rim				
pattern	Rim	1.92 (1.29–2.84)	0.001	1.87 (1.26–2.80)	0.002
Non-peripheral washout		1.24 (0.87–1.76)	0.229		
Arterial peritumoral enhancement		1.79 (1.22–2.63)	0.003		
Enhancing	Intact				
Capsule	Incomplete	1.47 (0.90–2.42)	0.124		
	Deficient	0.89 (0.45–1.75)	0.729		
Mosaic appearance		2.08 (1.46–2.97)	< 0.001	1.55 (1.04–2.31)	0.033
Intertumoral necrosis		1.76 (1.28–2.43)	0.001		
Intertumoral hemorrhage		2.11 (1.48–2.99)	< 0.001		
Resection extent	Minor				
	Major	1.28 (0.74–2.22)	0.380		
Grade	1				
	2	2.92 (0.41–20.93)	0.286		
	3	3.29 (0.45–23.79)	0.239		
MVI	0				
	1	1.15 (0.80–1.65)	0.458		
	2	2.17 (1.43–3.29)	< 0.001		

*ADC* apparent diffusion coefficient, *ALBI* albumin–bilirubin score, *ALT* alanine aminotransferase, *AST* aspartate aminotransferase, *CI* confidence interval, *DCP* des-gamma-carboxyprothrombin, *DNA* deoxyribonucleic acid, *HBV* hepatitis B virus, *HR* hazard ratio, *MVI* microvascular invasion, *TBS* tumor burden score

The independent variables above were finally incorporated into the predictive model. For predicting recurrence within 2 years, the mean *C*-index of the model was 0.728 (95% CI: 0.726–0.729) in training sets and 0.716 (95% CI: 0.711–0.720) in validation sets. For predicting recurrence within 5 years, it was 0.674 (95% CI: 0.673–0.675) in training sets and 0.657 (95% CI: 0.653–0.660) in validation sets. The distribution of the *C*-index after cross-validation is shown in Supplementary Fig. 2.

**Fig. 2:**
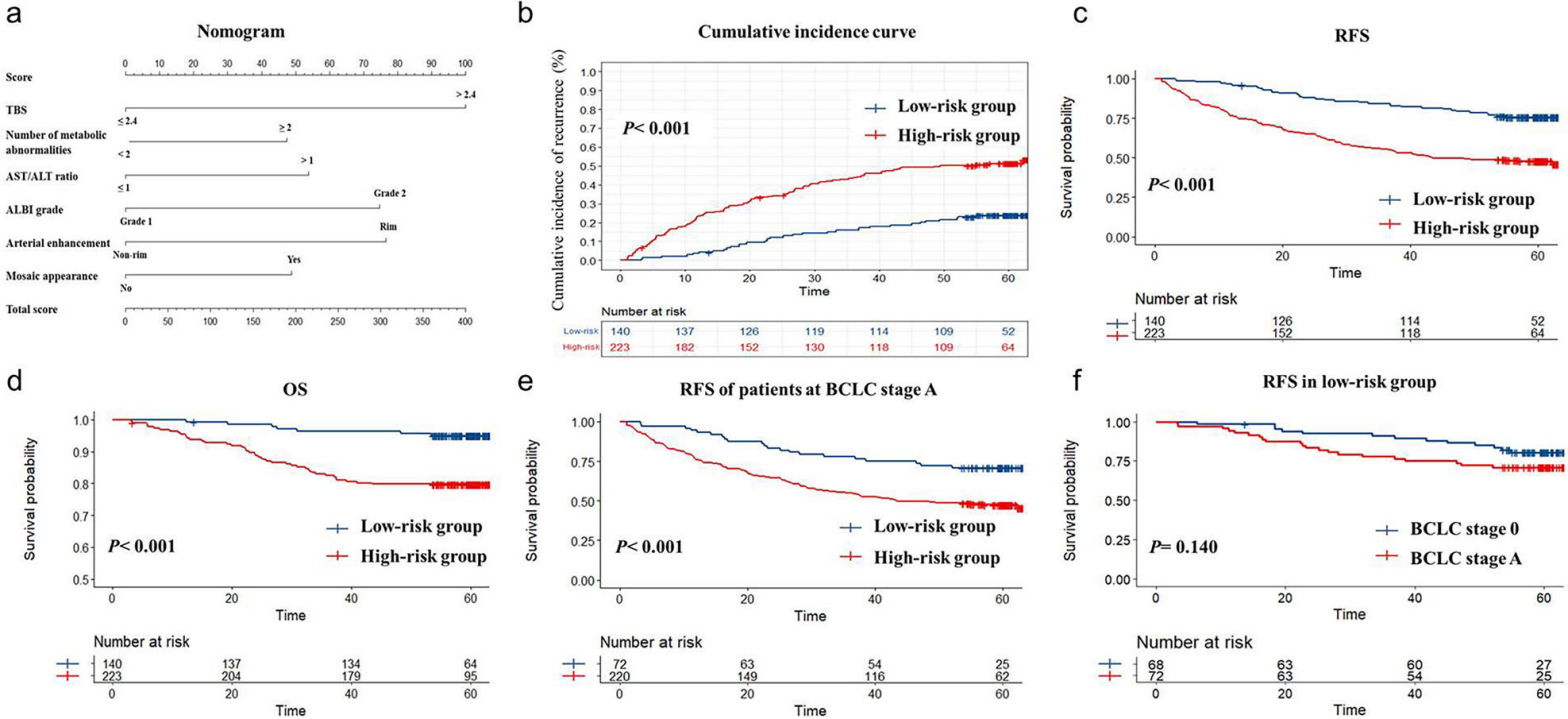
Internal validation of the clinical-radiological model. **a** Nomogram of the clinical-radiological model. **b** The cumulative incidence rates of recurrence, (**c**) recurrence free survival (RFS), and (**d**) overall survival (OS) between the low- and high-risk groups in the internal cohort; RFS (**e**) between the low- and high-risk groups in patients at BCLC stage A; **f** between BCLC stages 0 and A in the low-risk group. ALBI, albumin–bilirubin score; ALT, alanine transaminase; AST, aspartate transaminase; CNLC, China liver cancer; TBS, tumor burden score

The formula for the risk score was as follows (Fig. 2a):

Risk score = 100 × (TBS > 2.4) + 47.3 × (number of metabolic abnormalities ≥ 2) + 53.8 × (AST/ALT > 1) + 74.7 × (ALBI grade 2) + 76.6 × (arterial rim enhancement) + 48.7 (mosaic appearance).

The optimal cutoff value determined by the maximum Youden index was 148.7. The internal cohort population was then divided into the low-risk group with risk scores ≤ 148.7 (*n* = 140) and the high-risk group with risk scores > 148.7 (*n* = 223). As shown in Fig. 2b, the high-risk group had higher cumulative incidences of recurrence (*p* < 0.001). There was a significant difference in RFS between the low-risk and high-risk groups (*p* < 0.001, Fig. 2c), with a 2-year RFS of 87.4% and a 5-year RFS of 75.5% in the low-risk group, compared to 64.8% and 46.8% in the high-risk group, respectively. Additionally, there was also a significant difference in OS rates (*p* < 0.001, Fig. 2d). The 2-year and 5-year OS rates were 97.8% and 95.0% in the low-risk group, compared to 88.3% and 79.7% in the high-risk group. Multivariate Cox regression showed that after adjusting for the tumor grade, the risk score stratification was also associated with the risk of recurrence (HR = 2.76, 95% CI: 1.87–4.09, *p* < 0.001).

**Fig. 3:**
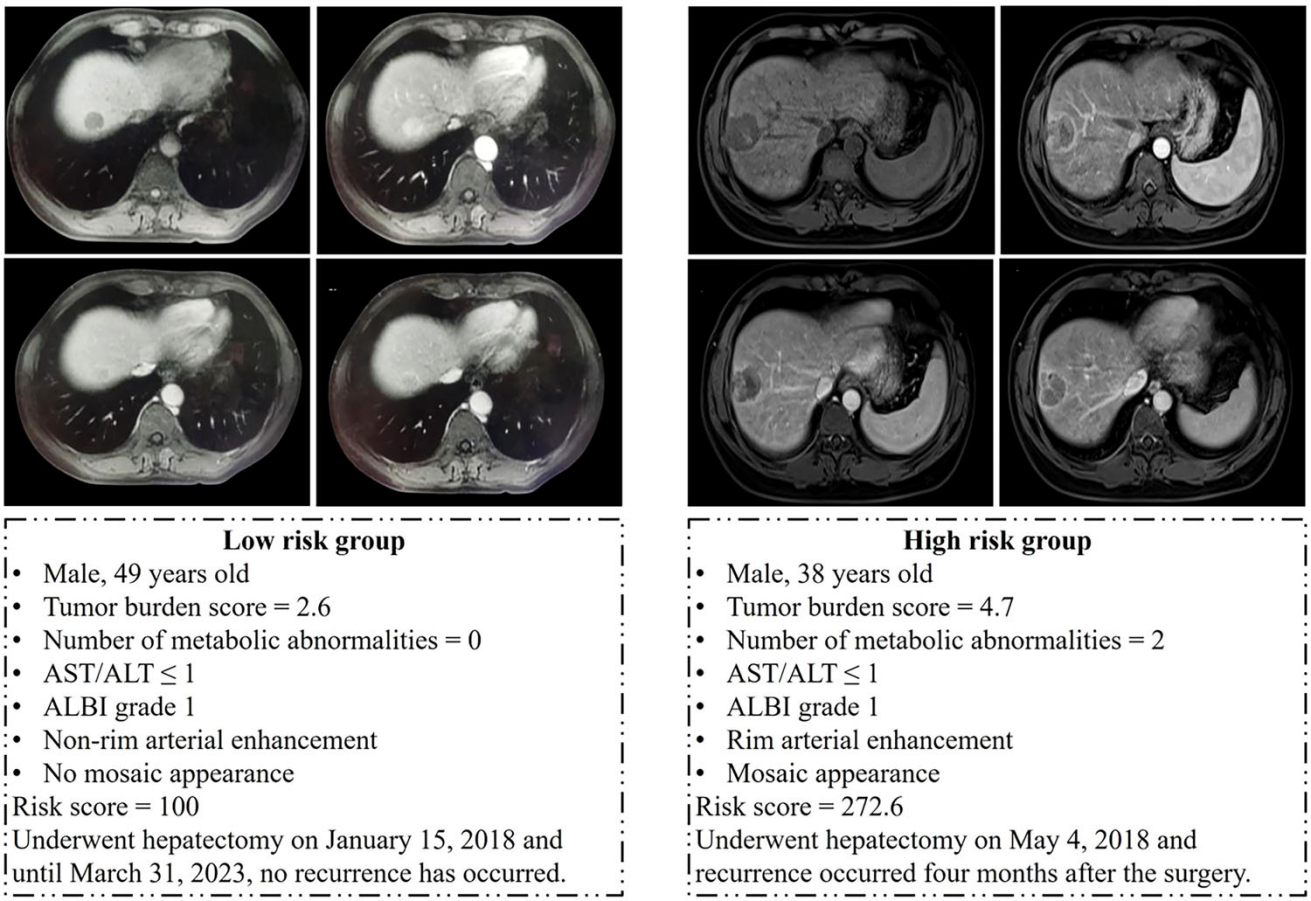
Examples of the low- and high-risk groups. Pre-contrast T1-weighted (top left), arterial phase (top right), portal venous phase (bottom left), and delayed phase (bottom right). ALBI, albumin–bilirubin score; ALT, alanine transaminase; AST, aspartate transaminase

Most patients of BCLC stage 0 were in the low-risk group (68/71). There was a significant difference in RFS between low- and high-risk groups in patients within BCLC stage A (*p* < 0.001, Fig. 2e), while there was no significant difference in RFS between BCLC stage 0 and stage A patients within the low-risk group (*p* = 0.140, Fig. 2f). Figure 3 showed two examples in the low-risk and high-risk groups.

**Fig. 4:**
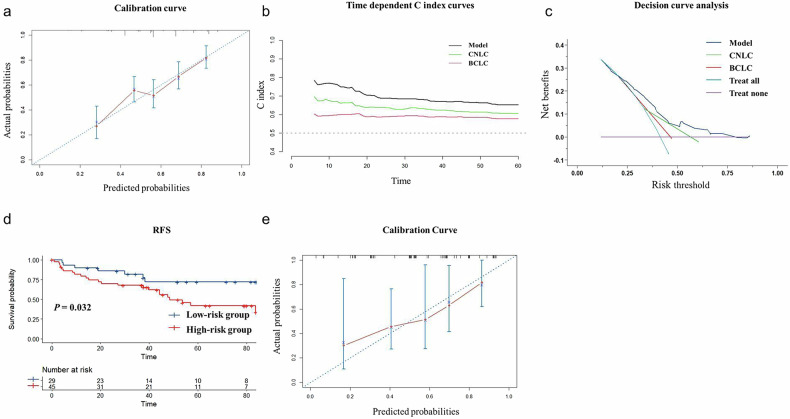
Predictive performance and external validation. **a** Calibration curve. **b** Time-dependent *C*-index curves and (**c**) decision curves of the clinical-radiological model, BCLC and CNLC staging systems. **d** RFS between the low- and high-risk groups in the external cohort. **e** Calibration curves. BCLC, Barcelona Clinic Liver Cancer; CNLC, China liver cancer; RFS, recurrence free survival

Figure 4a presented the model’s calibration curve, showing good model consistency. Figure 4b displayed the time-dependent *C*-index curves, demonstrating superior predictive performance compared to the BCLC and CNLC staging systems in very early or early-stage HCC. The 1-year, 2-year, and 5-year *C* indices of the predictive model were higher than those of the BCLC (*p* < 0.001 for all) and CNLC (*p* < 0.001 for all) staging systems for those patients. The model exhibited better net benefit compared to the existing clinical staging systems (Fig. 4c).

### External validation

The median follow-up time for the external cohort was 65.0 months (IQR: 38.9–83.9 months). Among patients with metabolic abnormalities in the external cohort, 9 patients (34.6% of patients with elevated blood glucose) received antidiabetic therapy, 18 patients (50.0% of patients with elevated blood pressure) received antihypertensive medications, and 2 patients (8.3% of patients with atherogenic dyslipidemia) received lipid-modifying agents. The clinical-radiological model achieved a *C*-index of 0.710 (95% CI: 0.602–0.855) for predicting recurrence within 2 years, and 0.683 (95% CI: 0.594–0.798) for predicting recurrence within 5 years. The RFS for low-risk and high-risk groups showed a significant difference (*p* = 0.032), with a 2-year RFS of 85.9% and a 5-year RFS of 72.1% for the low-risk group, compared to 68.7% and 41.9% for the high-risk group (Fig. 4d). Figure 4e displayed the calibration curve of the model in the external validation cohort.

## Discussion

According to our study, the number of metabolic abnormalities was dose-dependently related to the risk of postoperative recurrence in patients with HBV-related HCC at BCLC stages 0-A. Furthermore, we developed a clinical-radiological prediction model including the metabolic burden. Patients were stratified into two risk strata, with the predicted high-risk patients demonstrating worse survival. The model demonstrated good consistency, with higher predictive efficacy and better clinical net benefit compared to the existing BCLC and CNLC staging systems for very early/early-stage HCCs.

Previous studies have suggested that the number of metabolic abnormalities was dose-dependently related to the risk of HCC development [23–26]. Similarly, our study verified that the number of metabolic abnormalities was also dose-dependently associated with the risk of postoperative recurrence in HBV-related HCC. The metabolic abnormalities evaluated in this study were major components of metabolic syndrome, with insulin resistance being the central link [34, 35]. Insulin resistance may be a common cause of the increased risk of postoperative recurrence. Previous studies indicated that when insulin binds to its receptor, it initiates the sequence of phosphorylation events that lead to activation of the catalytic activity of phosphoinositide 3-kinase, and when chronically activated, this signal pathway can drive malignant transformation and tumor progression [17, 36, 37]. Oxidative stress, chronic inflammation, and alterations in the gut microbiota also collectively drive metabolic abnormalities toward hepatocarcinogenesis [17]. Previous studies indicated that medications like metformin and statins, commonly used for metabolic conditions, are associated with significantly reduced HCC recurrence and improved survival after resection [38–40]. Meanwhile, like previous studies [41, 42], our study also considered ALBI grading and AST/ALT ratio as key parameters for postoperative recurrence prediction.

The radiological variables included in the model were TBS, arterial rim enhancement, and mosaic appearance. TBS was proposed by integrating both the number and the maximum diameter of tumors, which presented superior postoperative recurrence prediction capability [28]. Arterial rim enhancement, a type of targetoid morphology related to the LR-M category, was reported to be an independent predictor of proliferative HCC, which was associated with aggressive clinical and pathological characteristics [43, 44]. Meanwhile, the mosaic appearance implied intratumoral pathological heterogeneity, which indicated more aggressive biological behavior and was associated with a higher tumor grade of HCC [45]. It was also verified as an important predictor of postoperative recurrence, consistent with our results [46].

The model constructed in our study had a good predictive efficacy for postoperative recurrence in both internal and external validation cohorts, indicating its satisfying generalization ability and robustness. Another important finding was that, for patients at very early or early stages, our model demonstrated superior predictive performance compared to the existing clinical staging systems of BCLC and CNLC, which may offer valuable advice for treatment planning and postoperative follow-up arrangements clinically. Moreover, the clinical and radiological variables incorporated in the model were easily obtainable through the routine clinical practice, without relying on complex software or post-processing techniques.

Our study had several limitations. Firstly, this study was retrospective, and the systematic errors caused by selection bias and recall bias were unavoidable. Notably, our cohort exclusively comprised surgical patients with BCLC stages 0-A. Future studies are warranted to validate the impact of metabolic abnormalities in more advanced-stage patients. Secondly, the study population in the external validation cohort was limited, and the distribution of tumor grade differed between the internal and external validation cohorts. However, according to our results, the risk score was associated with recurrence regardless of the tumor grade. While this cohort included patients from both temporal and geographical validation, larger multi-center studies are needed for definitive generalizability. Thirdly, as a retrospective study, the metabolic abnormalities evaluated in this study were based on the patients’ preoperative baseline levels, the impact of dynamic changes in metabolic parameters and the long-term effects of relevant treatments on recurrence could not be assessed. Large-scale prospective studies are warranted to validate these aspects.

## Conclusions

The metabolic burden-based clinical-radiological model was valuable in predicting postoperative recurrence, with better prognostic stratification potential compared to existing clinical staging systems in the very early/early-stage of HCCs.

## Supplementary information

ELECTRONIC SUPPLEMENTARY MATERIAL

## Data Availability

The datasets used and/or analyzed during the current study are available from the corresponding author on reasonable request.

## Abbreviations

aHR: Adjusted hazard ratio
ALBI: Albumin–bilirubin score
ALT: Alanine transaminase
AST: Aspartate aminotransferase
BCLC: Barcelona clinic liver cancer
BMI: Body mass index
CI: Confidence intervals
CNLC: China liver cancer staging
HBV: Hepatitis B virus
HCC: Hepatocellular carcinoma
HR: Hazard ratio
LI-RADS: Liver imaging reporting and data system
TOL: Tolerance
VIF: Variance inflation factor
