# Supplementary material for: Metabolic burden-based clinical-radiological model for predicting postoperative recurrence of hepatitis B-related hepatocellular carcinoma
Source: Insights Imaging. 2026 Jan 5;17:5. doi: 10.1186/s13244-025-02183-3 (PMC12770151; doi:10.1186/s13244-025-02183-3)
Supplement: Supplementary file 1 — ELECTRONIC SUPPLEMENTARY MATERIAL [file 13244_2025_2183_MOESM1_ESM.pdf]

Metabolic burden-based clinical-radiological model for predicting postoperative recurrence of hepatitis B-related hepatocellular carcinoma

ELECTRONIC SUPPLEMENTARY MATERIAL

Insights Imaging (2025) Zheng B, Wagn H, Xiao Y, et al.

Supplementary Table 1 MRI sequence parameters

	Parameter	T1-weighted IP and OP imaging	Contrast enhanced T1-weighted imaging	T2-weighted imaging	Diffusion weighted imaging
Magnetom Aera 1.5T	Repetition time (ms)	6.87	4.36	2000	2291
	Echo time (ms)	2.38/4.76	2.0	94.0	48.9
	Field of view (mm^2)	380×278	380×297	380×308	380×280
	Matrix	320×240	320×240	320×224	144×100
	Section thickness (mm)	4.0	3.0	5.5	6.0
	Gap (mm)	0	0	1.1	1.2
Prisma 3.0T	Repetition time (ms)	3.97	3.10	587	5000
	Echo time (ms)	1.29/2.52	1.23	103	65.0
	Field of view (mm^2)	380×285	380×309	380×344	380×310
	Matrix	320×288	320×288	320×320	130×130
	Section thickness (mm)	3.0	3.0	6.0	5.0
	Gap (mm)	0	0	1.2	1.2

Supplementary Table 2 Diagnostic criteria for metabolic abnormalities

Variables	Assessment	Number in internal cohort	Number in external cohort
Overweight or obese	Overweight was defined as BMI= 24.0-27.9kg/m^2 and obesity was defined as BMI≥ 28.0 kg/m^2.	186	31
Elevated blood pressure	Systolic blood pressure≥ 130mmHg and/or diastolic blood pressure≥ 85mmHg, or current use of antihypertensive medications.	234	36
Elevated blood glucose	Fasting blood glucose≥ 5.6mmol/L and/or glycated hemoglobin≥ 6.5%, or current antidiabetic therapy.	136	26
Atherogenic dyslipidemia	Hypertriglyceridemia: triglycerides≥ 1.7mmol/L, or current lipid-lowering treatment;<br>Low high-density lipoprotein cholesterol: high-density lipoprotein cholesterol< 1.0 mmol/L in male and< 1.3 mmol/L in female, or current lipid modifying agents.	136	24

Supplementary Table 3 Interobserver agreements of assessing imaging characteristics.

**Variables**	**κ/ICC**	**95% CI**	***P***
Maximum diameter	0.850	0.817-0.877	< 0.001
Location	0.731	0.619-0.842	< 0.001
Margin	0.755	0.547-0.962	< 0.001
Signal homogeneity	0.868	0.801-0.934	< 0.001
Arterial enhancement pattern	0.882	0.817-0.946	< 0.001
Non-peripheral washout	0.783	0.716-0.850	< 0.001
Peritumoral enhancement	0.775	0.691-0.859	< 0.001
Enhancing capsule	0.737	0.680-0.794	< 0.001
Mosaic appearance	0.792	0.727-0.857	< 0.001
Intertumoral necrosis	0.880	0.821-0.939	< 0.001
Intertumoral hemorrhage	0.858	0.795-0.921	< 0.001

Abbreviation: CI, confidence interval; ICC, Intra-class correlation coefficient.

Supplementary Table 4 Collinearity diagnostics

Variables	TOL	VIF
Gender	0.895	1.118
TBS	0.651	1.536
HBV DNA	0.870	1.149
Number of metabolic abnormalities	0.903	1.107
Log DCP	0.647	1.546
AST/ALT ratio	0.821	1.218
ALBI grade	0.904	1.106
Location	0.908	1.102
Margin	0.803	1.246
Signal homogeneity	0.550	1.819
Arterial enhancement pattern	0.758	1.319
Peritumoral enhancement	0.850	1.177
Enhancing capsule	0.856	1.168
Mosaic appearance	0.527	1.897
Intertumoral necrosis	0.498	2.008
Intertumoral hemorrhage	0.654	1.528
MVI	0.741	1.349

Abbreviation: ALT, alanine aminotransferase; AST, aspartate aminotransferase; ALBI, albumin-bilirubin; DCP, des-gamma-carboxyprothrombin; DNA, deoxyribonucleic acid; HBV, hepatitis B virus; TBS, tumor burden score; TOL, tolerance; VIF, variance inflation factor; MVI, microvascular invasion.

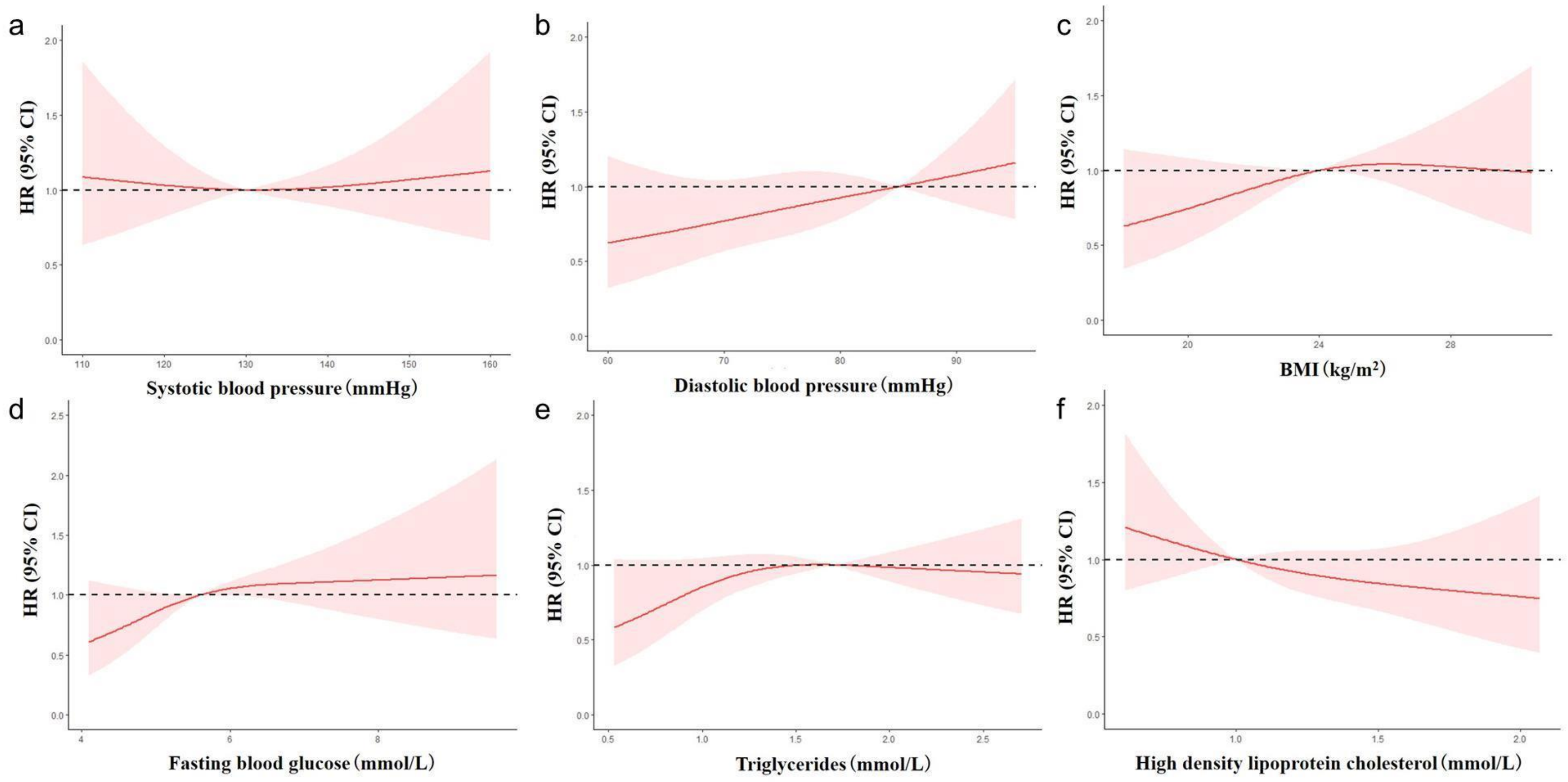

Supplementary Figure 1 Adjusted smooth hazard ratio (HR) curves for metabolic indicators. BMI, body mass index.

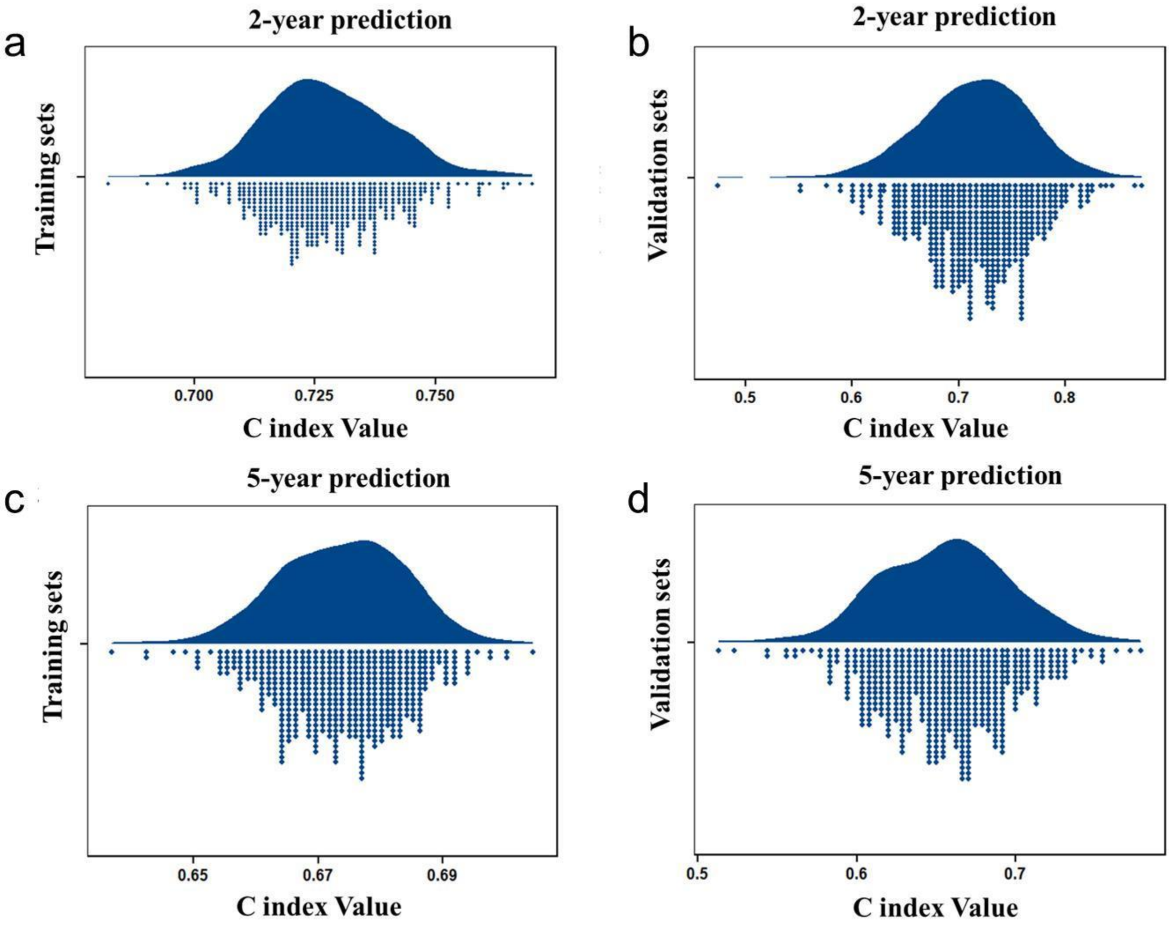

Supplementary Figure 2 5-fold 100 times cross validation. Distribution of C index for predicting recurrence within 2 years in (a) training and (b) validation sets, and within 5 years in (c) training and (d) validation sets.

Insights Imaging (2025) Zheng B, Wagn H, Xiao Y, et al.
